# CB_2_ receptor activation causes an ERK1/2-dependent inflammatory response in human RPE cells

**DOI:** 10.1038/s41598-017-16524-w

**Published:** 2017-11-23

**Authors:** M. Hytti, S. Andjelic, N. Josifovska, N. Piippo, E. Korhonen, M. Hawlina, K. Kaarniranta, T. J. Nevalainen, G. Petrovski, T. Parkkari, A. Kauppinen

**Affiliations:** 1 School of Pharmacy, University of Eastern Finland, Kuopio, Finland; 2 Department of Ophthalmology, School of Medicine, University of Eastern Finland, Kuopio, Finland; 3 Eye Hospital, University Medical Centre, Ljubljana, Slovenia; 4 Stem Cells and Eye Research Laboratory, Department of Ophthalmology, Faculty of Medicine, University of Szeged, Szeged, Hungary; 5 Department of Ophthalmology, Kuopio University Hospital, Kuopio, Finland; 6 Centre of Eye Research, Department of Ophthalmology and the Norwegian Center for Stem Cell Research, Oslo University Hospital, University of Oslo, Oslo, Norway

## Abstract

A chronic low-level inflammation contributes to the pathogenesis of age-related macular degeneration (AMD), the most common cause of blindness in the elderly in Western countries. The loss of central vision results from attenuated maintenance of photoreceptors due to the degeneration of retinal pigment epithelium (RPE) cells beneath the photoreceptor layer. It has been proposed that pathologic inflammation initiated in RPE cells could be regulated by the activation of type 2 cannabinoid receptors (CB_2_). Here, we have analysed the effect of CB_2_ activation on cellular survival and inflammation in human RPE cells. RPE cells were treated with the selective CB_2_ agonist JWH-133 in the presence or absence of the oxidative stressor 4-hydroxynonenal. Thereafter, cellular viability as well as the release of pro-inflammatory cytokines and potential underlying signalling pathways were analysed. Our results show that JWH-133 led to increased intracellular Ca^2+^ levels, suggesting that RPE cells are capable of responding to a CB_2_ agonist. JWH-133 could not prevent oxidative stress-induced cell death. Instead, 10 µM JWH-133 increased cell death and the release of proinflammatory cytokines in an ERK1/2-dependent manner. In contrast to previous findings, CB_2_ activation increased, rather than reduced inflammation in RPE cells.

## Introduction

Excessive inflammatory processes in human retinal pigment epithelial (RPE) cells are associated with the development of age-related macular degeneration (AMD)^1,2^, the leading cause of visual impairment in the elderly in the Western world^3^. RPE cells form a single-cell layer located at the posterior part of the eye between the choroid and the photoreceptors, and are vital for the survival and the functionality of rods and cones. They regulate the visual cycle as well as the transport of nutrients from the choroid to the photoreceptors and the removal of waste products away from the retina^4,5^. RPE cells also renew photoreceptors by degrading their outer segments in the process called heterophagy, participate in the formation of the blood-retinal barrier, and maintain the ion balance and immune responses in the retina^1,6–9^. Dysfunction of the RPE leads to the degeneration and death of photoreceptors, causing the distinctive loss of central vision in AMD^4,5^ (reviewed in^6,10^).

One protein receptor potentially capable of modulating inflammatory responses is the cannabinoid receptor type 2 (CB_2_). The G-protein-coupled receptor is one of the two receptors targeted by pharmacologically active, plant-derived cannabinoids as well as the body’s own endocannabinoids^11,12^. Another cannabinoid receptor is CB_1_, which is predominantly expressed in the central nervous system (CNS)^13^. Along with neuroprotective effects, the CB_1_ receptor mediates the psycho-active effects of cannabinoids, such as increased appetite, hallucinations, and antiemesis^11,14^. In contrast, the CB_2_ receptor is expressed predominantly in the periphery, especially on immune cells, and has been linked to many of the beneficial, anti-inflammatory effects of cannabinoids^13^.

Specific agonists of CB_2_ have been developed to facilitate the studies of the receptor’s effects and to avoid side-effects associated with CB_1_ activation^15,16^. Studies utilizing these activators found that CB_2_ activation reduced the production of IL-6 in lipopolysaccharide (LPS)-treated murine macrophages and reduced the severity of collagen-induced arthritis in mice^17^. However, many effects of CB_2_ receptor agonists have been found to depend on the studied cell type, the culture conditions, and the agonist used^13^. Schmöle *et al*. found that the knock-out of CB_2_ reduced the release of IL-6 from primary microglia upon LPS stimulation^18^. At the same time, others have found the activation of CB_2_ to be anti-inflammatory^19,20^.

CB_2_ is expressed by RPE cells^21^ and endocannabinoid levels are increased in the eyes of AMD patients^22^. In one study, CB_2_ activation was found to reduce the hydrogen peroxide-induced death of ARPE-19 and primary human RPE cells, suggesting a beneficial effect of CB_2_ receptor activation in the treatment or the prevention of AMD^21^. However, studies on the role of CB_2_ receptor in human RPE cells are scarce and data about its effects on inflammation in other cells are inconsistent.

Here, we describe that JWH-133, a direct agonist of the CB2 receptor, increases the release of pro-inflammatory cytokines IL-6 and IL-8 from human RPE cells. This effect was associated with augmented ERK1/2 activation and increased intracellular Ca^2+^ levels.

## Results

### JWH-133 does not protect cells from HNE-induced cytotoxicity

**Figure 1:**
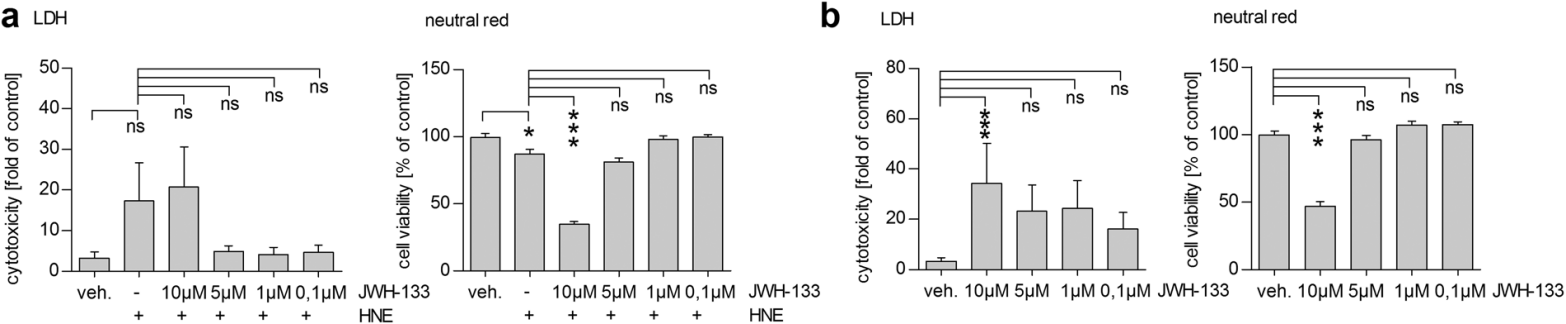
JWH-133 does not protect ARPE-19 cells from HNE-induced cytotoxicity, but is toxic at the concentration of 10 µM. Analysis of cell death and cell viability by the LDH and neutral red assay, show a moderate decrease in cell viability after an exposure to HNE (**a**), which could not be prevented by any of the studied concentrations of JWH-133. Instead, 10 µM JWH-133 was toxic to both untreated (**b**) and HNE-treated ARPE-19 cells (**a**). Results are shown as mean ± SEM and combined from 3-6 independent repetitions with 4–6 parallels per group. ns denotes not statistically significant, *denotes *P* < 0.05, ***denotes *P* < 0.001; Mann–Whitney *U*-test.

One previous report indicated that the activation of the CB_2_ receptor in ARPE-19 cells is protective against oxidative stress-related cell death caused by hydrogen peroxide^21^. We have previously shown that the reactive aldehyde 4-hydroxynonenal (HNE), an abundant source of oxidative stress in the retina *in vivo*, increases cytotoxicity in human RPE cells^23^. Here, we tested whether the activation of CB_2_ with JWH-133 would protect ARPE-19 cells from the cytotoxicity induced by HNE. We found that none of the studied concentrations of JWH-133 were able to prevent HNE-induced cytotoxicity (Fig. 1a). Instead, 10 µM JWH-133 proved to be cytotoxic, reducing the cell viability an additional 53% when compared to HNE-treatment alone (Fig. 1a). In both LDH and neutral red assays, 10 µM JWH-133 caused significant cytotoxicity also without HNE treatment (Fig. 1b).

### JWH-133 activates inflammation in RPE cells

**Figure 2:**
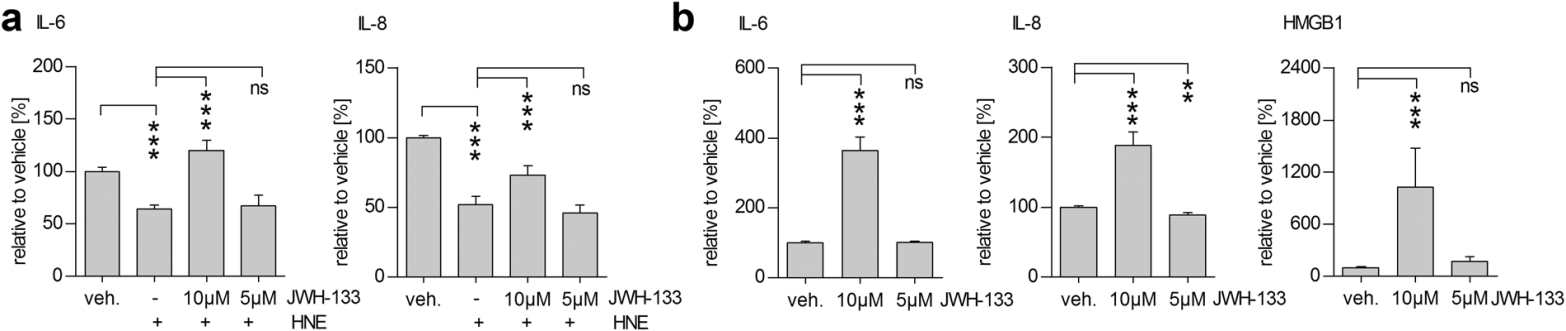
JWH-133 stimulated an inflammatory response in ARPE-19 cells. Treatment of cells with 10 µM JWH-133 led to increased secretion of IL-6, IL-8 to the cell culture medium after HNE treatment (**a**). This increase was comparable also in the absence of HNE, where JWH-133 by itself increased IL-6, IL-8, and HMGB1 levels (**b**). Results are shown as mean ± SEM and combined from 3 independent repetitions with 3–4 parallels per group. ns denotes not statistically significant, ***denotes *P* < 0.001; Mann–Whitney *U*-test.

In conjunction with the increased cytotoxicity induced by 10 µM JWH-133, it additionally increased the secretion of pro-inflammatory cytokines IL-6 and IL-8 from ARPE-19 cells exposed to HNE (Fig. 2). In accordance with our previous results^23,24^, HNE alone decreased the release of IL-6 and IL-8 from ARPE-19 cells, most likely due to the inhibition of nuclear factor κB (NF-κB). Despite the decreased IL-6 and IL-8 levels following the HNE treatment, an exposure of RPE cells to 10 µM JWH-133 still raised the cytokine levels by 62% and 64%, respectively (Fig. 2a). In cells that were not subjected to oxidative stress, 5 μM, JWH-133 slightly decreased IL-8 levels but had no effect on either IL-6 or HMGB1 (Fig. 2b). 10 µM JWH-133 alone significantly increased the levels of IL-6 and IL-8, as well as those of HMGB1 (Fig. 2b).

### JWH-133 induces a calcium response in ARPE-19 cells

**Figure 3:**
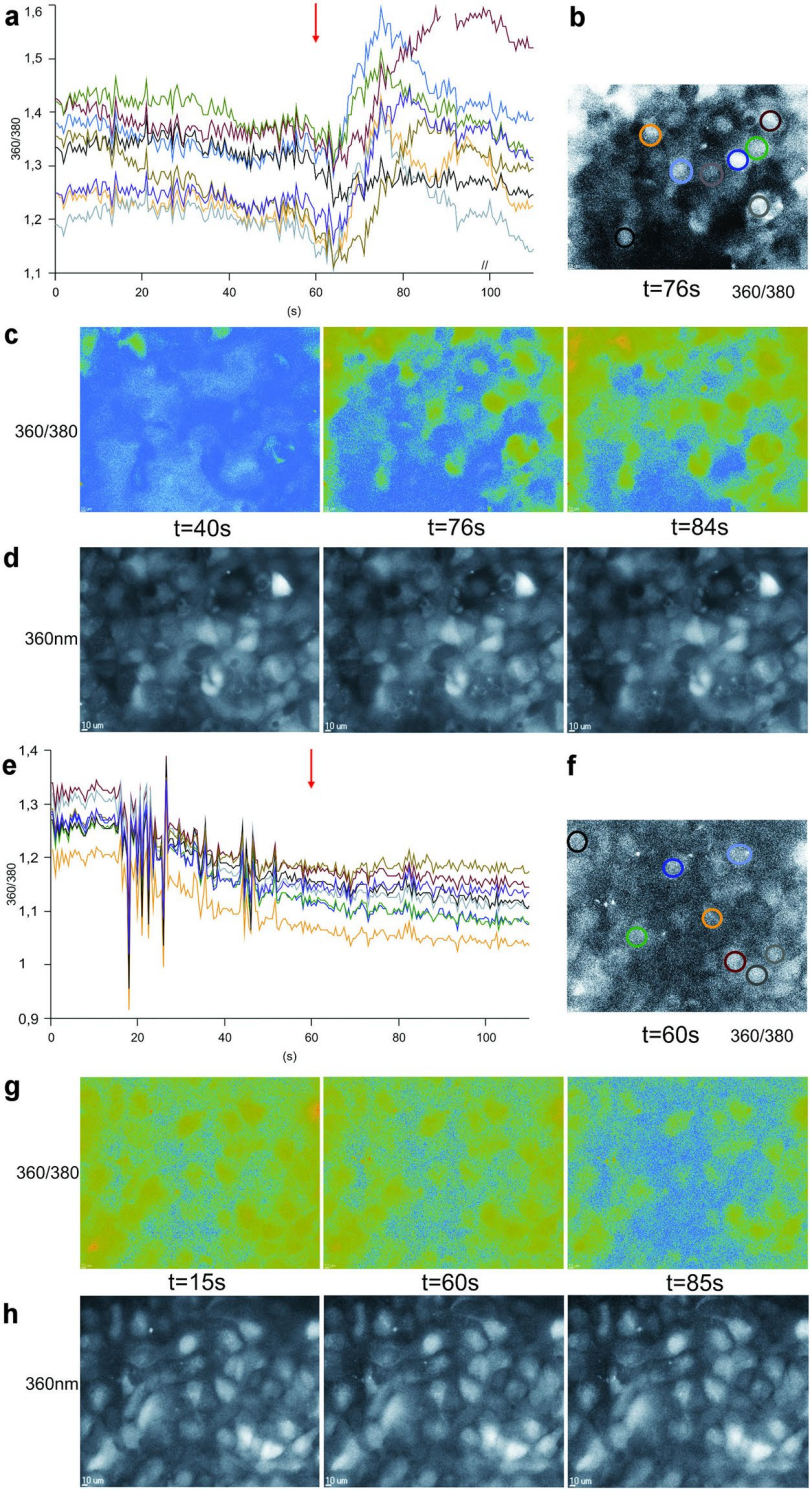
JWH-133 treatment leads to an increase in intracellular calcium levels. A stimulation of confluent ARPE-19 cell cultures with 5 µM JWH-133 (**a**–**d**) or 1% BSA (**e**–**h**), which served as control. [Ca^2+^]i increased after stimulation with 5 µM JWH-133, which was added at the 60 s timepoint (red arrows), followed by a return to pre-stimulation levels (**a**). 1% BSA did not cause a similar increase in [Ca^2+^]i (**e**). The 360 ⁄ 380 ratios are proportional to the changes in intracellular calcium. The timecourse of the changes to the 360/380 ratios (**a**,**e**) are coloured to correspond to colour coded single ARPE-19 cells (**b**,**f**). 360/380 ratio images of selected time points, both before and after the stimulation illustrate these changes in [Ca^2+^]i (**c**,**g**). Low ratio values are represented in blue, while green represents high ratio values. Cell morphology was not influenced by JWH-133 treatment, as illustrated by the raw 360 nm fluorescent images (**d**,**h**).

Activation of the CB_2_ receptor has previously been shown to increase intracellular Ca^2+^ ([Ca^2+^]_i_) levels in human embryonic kidney cells^25^. In our experiments, ARPE-19 cells exposed to 5 µM JWH-133 showed increased intracellular Ca^2+^ levels, which subsequently returned to the stationary state (Fig. 3a–d). No significant changes in ARPE-19 cell morphology were observed before or after the JWH-133 treatment (Fig. 3d). A control stimulation with 1% bovine serum albumin (BSA) did not increase [Ca^2+^]_i_ levels in ARPE-19 cells (Fig. 3e–h).

### JWH-133-induced inflammation is accompanied by increased ERK1/2 phosphorylation

**Figure 4:**
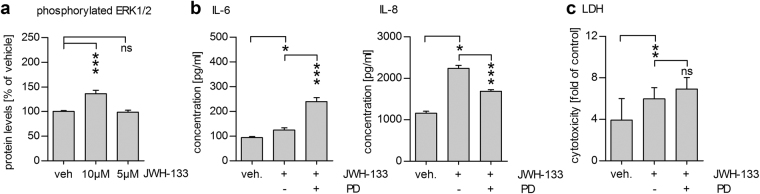
The inflammatory reaction caused by JWH-133 is related to ERK1/2 activation. Treatment of ARPE-19 cells with 10 µM JWH-133 led to increased ERK1/2 phosphorylation (**a**) alongside the increase in IL-6 and IL-8 levels (**b**). Inhibition of ERK1/2 signalling with the MEK1/2 inhibitor PD98059 (PD) led to decreased IL-8 release (**b**) without an increase in toxicity (**d**). Surprisingly, ERK1/2 inhibition led to increased IL-6 levels (**b**). Results are shown as mean ± SEM and combined from 3 independent repetitions with 2–4 parallels per group. ns denotes not statistically significant, *denotes *P* < 0.05, **denotes *P* < 0.01, ***denotes *P* < 0.001; Mann–Whitney *U*-test.

After observing that JWH-133 increased the release of pro-inflammatory cytokines from RPE cells, we next examined the phosphorylation status of ERK1/2, which has previously been associated with CB_2_ receptor activation^26,27^ In our experiments, 10 µM JWH-133 increased the phosphorylation of ERK1/2 in ARPE-19 cells (Fig. 4a). Additionally, the inhibition of ERK1/2 phosphorylation with PD98059 reduced the JWH-133-induced secretion of IL-8 by 25% (Fig. 4b). Controversially, ERK1/2 inhibition led to increased release of IL-6 from ARPE-19 cells (Fig. 4b). Inhibition of ERK1/2 had no effect on the cellular viability measured by the LDH assay (Fig. 4d).

### Results obtained with the ARPE-19 cell line are repeatable in primary human RPE cells

**Figure 5:**
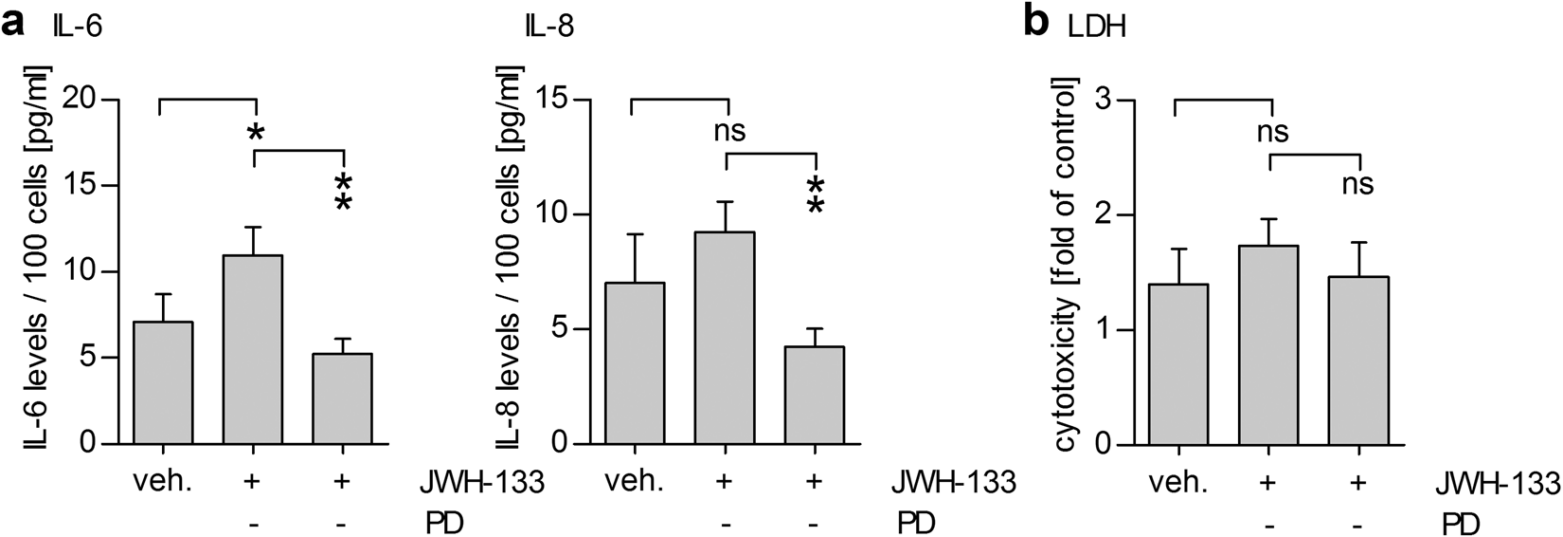
10 µM JWH-133 causes an ERK1/2-dependent inflammatory reaction in primary human RPE cells. Treatment of human primary RPE cells with 10 µM JWH-133 led to increased IL-6 and IL-8 secretion that was significantly reduced by PD98059 (PD) treatment. (**a**) PD98059 and JWH-133 were not toxic to primary human RPE cells (**b**). Results are shown as mean ± SEM and combined from independent repetitions using primary RPE cells from 5 separate donors with 1–2 parallel wells per treatment group per donor. ns denotes not statistically significant, *denotes *P* < 0.05, **denotes *P* < 0.01; Mann–Whitney *U*-test.

Repetition of our experiments in unpassaged hRPE cells also showed increased IL-6 and IL-8 secretion after an exposure to 10 µM JWH-133 (Fig. 5a). Inhibition of ERK1/2 with PD98059 decreased the levels of IL-6 and IL-8 by 52% and 54% respectively, efficiently reducing the levels of the inflammatory cytokines to control values (Fig. 5a). Neither JWH-133 treatment nor the addition of PD98059 was toxic to the studied primary RPE cells (Fig. 5b), which is in line with our previous findings that unpassaged primary hRPE cells are more resistant to cell death than ARPE-19 cells^28^.

## Discussion

The CB_2_ receptor is predominantly expressed by immune cells^12^. Its potential to modulate the immune response might be beneficial in the treatment of diseases associated with chronic low-level inflammation, such as atherosclerosis, diabetes, and AMD^1,29^. The CB_2_ receptor is highly inducible, and its expression increases strongly when microglia and other immune cells become activated in response to inflammatory stimuli^12^. CB_2_ receptor activation by a specific agonist could potentially control inflammatory responses and delay or prevent the onset of disease. The finding that CB_2_ receptors are expressed by RPE cells and that their activation protected these cells from oxidative stress-induced damage led to the suggestion that CB_2_ activation might be a possible new treatment strategy for AMD^21^.

Wei *et al*. were the first to show that the activation of CB_2_ could protect RPE cells from hydrogen peroxide-induced cell death^21^. In contrast, we found that the CB_2_ agonist JWH-133 had no protective effect on RPE cell survival after an exposure to the reactive aldehyde HNE. HNE is a product of lipid peroxidation and one of the most abundant oxidative stressors in the retina^30^. JWH-133 could not protect RPE cells from HNE-mediated death and even augmented the toxicity at a 10 µM concentration. At the same time, 10 µM JWH-133 increased the production of pro-inflammatory cytokines IL-6, IL-8, and HMGB1. This is in line with the results from Schmöle *et al*. who showed that CB_2_ deletion reduced the production of IL-6 in LPS-treated microglia, suggesting that CB_2_ can act as a pro-inflammatory factor under specific conditions^18^. CB_2_ knockout mice also showed diminished inflammation in response to severe induced sepsis compared to wild type mice^31^. However, multiple other groups have shown that the activation of CB_2_ leads to reduced, rather than increased production of pro-inflammatory cytokines. Activation of CB_2_ reduced the release of pro-inflammatory cytokines in LPS-induced uveitis^19^, and JWH-133 reduced the production of IL-6 in both, TNFα-stimulated fibroblast-like synoviocytes^20^ and a model of acute induced pancreatitis in mice^32^. Our results indicate that in RPE cells, CB_2_ activation causes increased inflammation, which could aggravate the pathogenesis of AMD.

CB_2_ modulation has resulted in contradictory findings in the past and the activation of CB_2_ is known to cause different reactions in cells depending on the choice of agonist, the activation status of the cells, or the cell type^12,13^. CB_2_ activation by the endocannabinoid 2-arachidonylglycerol induces migration in immune cells^33,34^, while other CB_2_ activators, both chemical and biological, are known to inhibit this migration^33,35^. The knockout of CB_2_ reduced the production of pro-inflammatory cytokines in LPS-stimulated CB_2_^−/−^ microglia and reduced the levels of cytokines and infiltrating microglia in the brain in an Alzheimer’s disease mouse model^18^. At the same time, in a model of controlled cortical impact injury, neuroinflammation was increased in CB_2_-knockout mice compared to wild-type animals^36^. It appears that both the activation and the inhibition of CB_2_ can exert proinflammatory, as well as anti-inflammatory effects depending on the context and the local circumstances of the employed disease model^12^.

**Figure 6:**
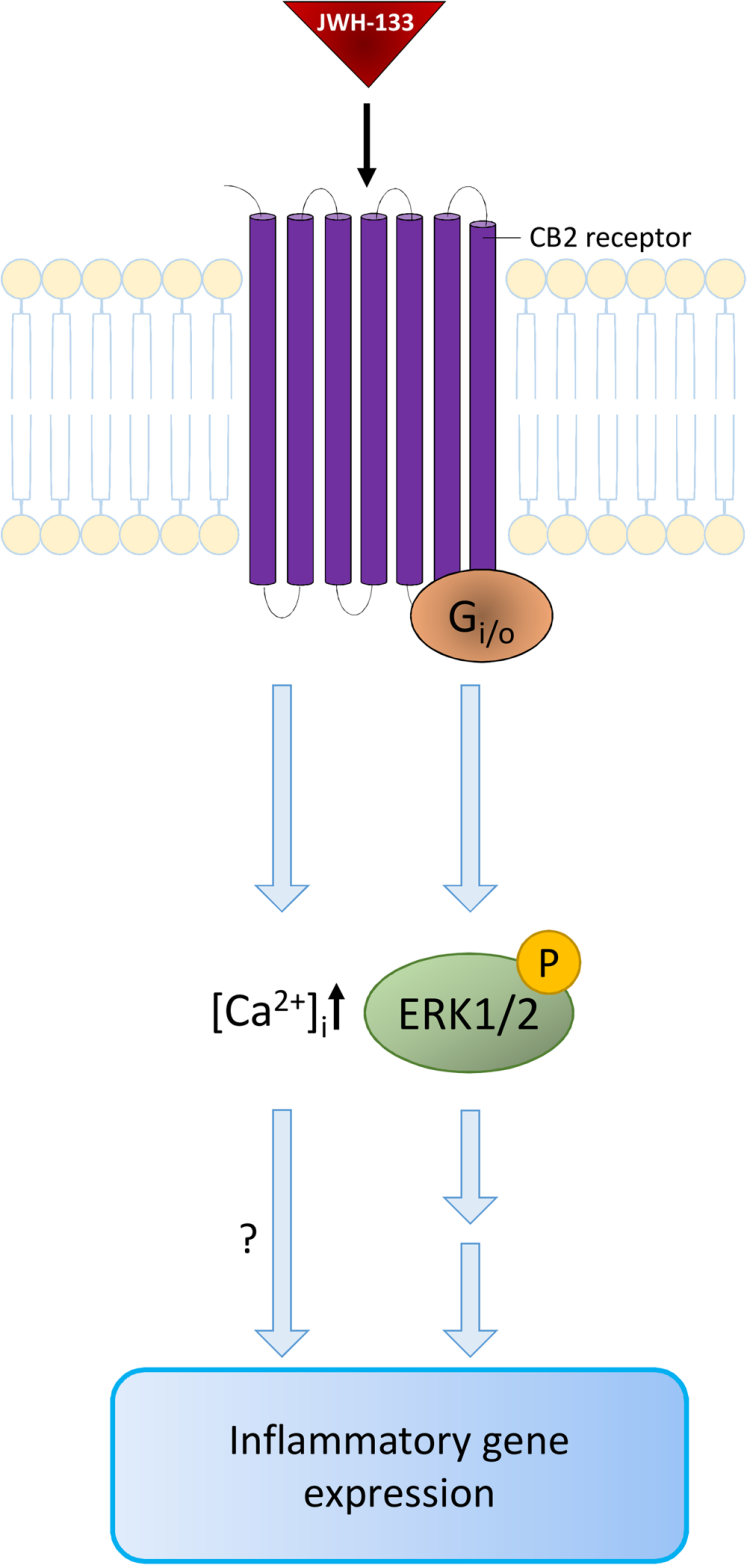
Suggested pathways activated by JWH-133 in ARPE-19 cells. JWH-133 activates the CB2 receptor, which leads to higher intracellular calcium levels and the phosphorylation of ERK1/2. MAPK signalling and possibly augmenting effects of calcium lead to an activation of RPE cell inflammation.

The complexity of CB_2_ receptor activation extends to the signalling pathways underlying its immunomodulatory effects. Research has shown that CB_2_ can influence different signalling pathways, including mitogen-activated protein kinase (MAPK) and cyclic adenosine monophosphate (cAMP) signalling^13^. To complicate matters, previous studies have suggested that CB2 activation can either increase^26,27^ or decrease^35,37^ the phosphorylation, and thereby the activity, of MAPK ERK1/2. CB_2_ activation has also been shown to increase intracellular Ca^2+^ levels^25^. In our study [Ca^2+^]_i_ is increased after the addition of JWH-133 to ARPE-19 cells, which is in line with previous observations^25^. Wei *et al*. were the first to report the expression of the CB_2_ receptor in ARPE-19 and primary human RPE cells, showing both, mRNA and protein expression of CB_2_^21^. Our results, indicating a Ca^2+^-response after JWH-133 treatment, provide further evidence for the presence of the CB_2_ receptor in RPE cells. Additionally, CB_2_ activation led to increased ERK1/2 phosphorylation, while the inhibition of ERK1/2 with a specific inhibitor reduced the JWH-133-induced secretion of IL-6 and IL-8 back to control levels in hRPE cells. This suggests that the activation of ERK1/2 is directly associated with the JWH-133-induced production of proinflammatory cytokines. We have previously shown that the inhibition of ERK1/2 can reduce inflammation in HNE-treated RPE cells^23^, which is in line with our current results. Additionally, increased [Ca^2+^]_i_ after CB_2_ activation could be involved in the release of pro-inflammatory cytokines. Calcium responses and ERK1/2 activation working in tandem, have been shown to be involved in the endothelin 1-induced production of IL-6 in human airway smooth muscle cells^38^, as well as in the production of IL-8 in oxysterol-treated monocytes^39^. Figure 6 illustrates a possible pathway of CB_2_ activation-linked inflammation in RPE cells. Future studies analysing the benefits of calcium channel blockers on JWH-133-induced inflammation in RPE cells could shed further light on the importance of the observed calcium response.

It is worth noticing that CB_2_ agonists are highly lipophilic compounds with a potential for unspecific binding^12^. However, increased intracellular Ca^2+^ levels coupled with an increased ERK1/2 phosphorylation is in line with previous findings related to CB_2_ activation^12,26,27^, indicating that our results are facilitated by CB_2_. Additional studies in different models, such as CB_2_-knockout mice could provide additional clarity concerning the role of CB_2_ in RPE cell-associated inflammation.

In summary, our results show that the activation of the CB_2_ receptor has detrimental effects on RPE cells, leading to increased pro-inflammatory cytokine production in an ERK1/2-dependent manner. Interestingly, endocannabinoid levels are increased in the retina of AMD patients^22^, which could suggest that CB_2_ activation plays an important role in the chronic inflammation underlying the disease. Further studies are necessary to fully elucidate the role of (endo)cannabinoids and their receptors in AMD. Nevertheless, our findings suggest that CB_2_ activation might contribute to AMD development rather than prevent it.

## Materials and Methods

### Cell culture

ARPE-19 cells were obtained from the American Type Culture Collection and were routinely kept in Dulbecco’s modified Eagle’s medium (DMEM) and nutrient mixture F-12 (1:1; Life Technologies, Carlsbad, CA, USA) supplemented with 10% HyClone fetal bovine serum (FBS; Thermo Fisher Scientific, Waltham, MA, USA), 100 U/ml penicillin, 100 µg/ml streptomycin, and 2 mM L-glutamine (Lonza, Basel, Switzerland). Cells were passaged every 3–4 days using 0.25% Trypsin-EDTA (Life Technologies, Carlsbad, CA, USA) and passages between 26 and 35 were used for experiments. The isolation and the use of primary human RPE cells were approved by, and performed in accordance with the guidelines of the ethics committee of the Medical and Health Science Center, University of Debrecen (DEOEC RKEB/IKEB Prot.No. 2745 -2008 and 3093 - 2010) and the Declaration of Helsinki. Cadaver eyes were enucleated and used for experiments only if they were deemed not suitable for corneal transplantation and after obtaining permission from the institutional research ethics committee (IREC). Hungary follows the EU Member States’ Directive 2004/23/EC on presumed consent practice for tissue collection and this is applicable to the samples collected for and used in the experiments approved by the IREC. Primary human RPE cells (hRPE) were collected from human donor eyes as described previously^40^. Cells were maintained without passaging in DMEM:F-12 (Sigma-Aldrich, St. Louis, MO, USA) supplemented with an antibiotic-antimycotic solution (Biosera, Boussens, France) and 10% FBS (Biosera, Boussens, France). Prior to experiments hRPE cells were assessed under the microscope to assure that the cells exhibited strong pigmentation and little to no contamination with fibroblast-like cells. Culture medium was changed for all cells twice per week during routine culture. All cells were incubated at +37 °C in a humidified atmosphere supplemented with 5% CO_2_.

### Cell treatments

ARPE-19 cells were treated on 12-well plates to which they were seeded at a density of 200.000 cells/ml/well and incubated for 48 h until confluent. hRPE cells were treated on fully confluent 24-well plates. All cells were washed once prior to treatments with serum-free maintenance medium supplemented with 1% bovine serum albumin (BSA; Roche, Basel, Switzerland). Cells were treated with the known CB_2_ agonist JWH-133 (Tocris Bioscience, Bristol, UK) and incubated for 24 h before the collection of serum and protein samples. To simulate high oxidative stress conditions, we treated part of the cells with 30 µM 4-hydroxynonenal (HNE; Calbiochem, San Diego, CA, USA) 15 minutes after the treatment with JWH-133. For inhibitor experiments, cells were pre-treated with the 50 µM concentration of the ERK1/2 inhibitor PD98059 (Cell Signaling Technologies, Danvers, MA, USA) for 30 minutes before the JWH-133 treatment. The concentration of PD98059 was based on previous dose-finding studies^23^. Untreated cells or cells exposed to equimolar amounts of dimethyl sulfoxide (DMSO; Sigma-Aldrich, St. Louis, MO, USA), the solvent of both PD98059 and JWH-133, served as controls. All experiments were performed at least three times with similar results.

### Enzyme-linked Immunosorbent Assays (ELISA)

Levels of IL-6 and IL-8 were determined from medium samples using specific BD OptEIA^TM^ human ELISA kits (BD, Franklin Lakes, NJ, USA). HMGB1 levels were measured using the HMGB1 ELISA kit from IBL international (Hamburg, Germany). Phosphorylated ERK1/2 levels were analysed from protein samples using the specific PathScan® Phospho-p44/42 MAPK (Thr202/Tyr204) Sandwich ELISA Kit (Cell Signaling Technologies, Danvers, MA, USA). Protein samples were collected by lysing cells in the mammalian protein extraction reagent (MPER) lysis buffer (Thermo Fisher Scientific, Waltham, MA, USA) after an initial wash with 1x phosphate buffered saline. Lysis of cells and all ELISAs were performed according to the manufacturers’ instructions.

### Cell Viability Assays

Cell viability was assessed with the lactate-dehydrogenase assay (LDH), the neutral red assay, or the 3-(4,5-dimethyldiazol-2-yl)-2,5-diphenyltetrazolium bromide (MTT, Sigma-Aldrich, St. Louis, MO, USA) assay. LDH was measured from medium samples according to the manufacturer’s instructions using the commercially available CytoTox96® Non-Radioactive Cytotoxicity Assay (Promega, Fitchburg, WI, USA). The neutral red assay was performed as described by Repetto *et al*.^41^ using 96-well plates to which cells were split at a density of 15000 cells/100 µl medium/well. The MTT assay was performed according to our laboratory’s standard protocol, which has been described before^42^.

### Calcium measurements

For Calcium imaging experiments, ARPE-19 cells were cultivated onto plastic glass bottom Petri dishes (Mattek Corp., USA; 3.5 cm in diameter) in 1:1 mixture of DMEM and Nutrient Mixture F12 medium (both obtained from Sigma, Steinheim, Germany) supplemented with 10% fetal calf serum (FCS) and 1% antibiotics (penicillin–streptomycin; Sigma, Steinheim, Germany) until complete attachment and full confluence in the presence of 5% CO_2_ at 37 °C. The Petri dish central part had a 14 mm glass bottom, with the remaining surface made from cell culture plastic and was poly-d-lysine coated. For monitoring of cytosolic free calcium concentrations ([Ca^2+^]_i_), the ARPE-19 cells were loaded with AM ester of Fura-2 (Fura-2 AM; Invitrogen–Molecular Probes, USA). For loading, Fura-2 AM in DMSO was diluted in 2 ml culture medium to a final concentration of 3 μM and added to the cells. Cells were loaded in the incubator at 37 °C for 1 hr. After loading, the ARPE-19 cells were washed twice for 7 min with culture medium. The Petri dish with the ARPE-19 cells was then mounted onto an inverted Zeiss Axiovert S 100 microscope (Carl Zeiss AG, Oberkochen, Germany). In order to evoke calcium responses in ARPE-19 cells, either 5 μM cannabinoid receptor agonist JWH-133 or 1% bovine serum albumin (BSA) alone, as a control, were applied. The application as well as its washout from the bath was driven by the hydrostatic pressure of a 35 cm of water column and controlled manually. Image acquisition was done with a 12-bit cooled CCD camera SensiCam (PCO Imaging AG, Kelheim, Germany). The software used for the acquisition was WinFluor (written by J. Dempster, University of Strathclyde, Glasgow, UK), while the optical objective used was 63x ⁄ 1,25 oil Plan-NeoFluar (Zeiss), and the light source was XBO-75W (Zeiss) Xe arc lamp. The light intensity was attenuated when necessary with grey filters with optical densities 0.5, 1 and 2 (Chroma Technology Corp., Bellows Falls, VT, USA). The excitation filters used and mounted on a Lambda LS-10 filter wheel (Sutter Instruments Co.) were 360 and 380 nm (Chroma). Excitation with the 360 nm filter (close to the Fura-2 isosbestic point) allowed observation of the cells’ morphology and of the changes in the concentration of the dye, irrespective of changes in [Ca^2+^]_i_, while the 360⁄380 nm ratio allowed visualization of the [Ca^2+^]_i_ changes in the cytoplasm. Image acquisition, timing and filter wheel operation were all controlled by WinFluor software via a PCI6229 interface card (National Instruments, Austin, TX, USA). Individual image frames were acquired every 500 ms resulting in frame cycles which were 1 second long (two wavelengths).

### Statistical Analysis

Results from ELISA and cell viability assays were analysed using GraphPad Prism (GraphPad Software Inc., San Diego, CA, USA). The data were tested for statistical significance by pairwise analysis of treatment groups using the Mann-Whitney *U*-test and a value of P < 0.05 was considered statistically significant.

### Data Availability

All data generated or analysed during this study are included in this published article.
